# The Relationship between Sense of Presence, Emotional Response, and Clinical Outcomes in Virtual Reality-Based Therapy for Treatment-Resistant Schizophrenia: An Exploratory Correlational Study

**DOI:** 10.3390/jpm14060614

**Published:** 2024-06-08

**Authors:** Elischa Augustin, Mélissa Beaudoin, Sabrina Giguère, Hind Ziady, Kingsada Phraxayavong, Alexandre Dumais

**Affiliations:** 1 Research Center of the University Institute in Mental Health of Montreal, Montreal, QC H1N 3V2, Canada; elischa.augustin@umontreal.ca (E.A.); melissa.beaudoin.1@umontreal.ca (M.B.); sabrina.giguere.2@umontreal.ca (S.G.); hind.ziady@umontreal.ca (H.Z.); 2 Department of Psychiatry and Addictology, Faculty of Medicine, University of Montreal, Montreal, QC H3T 1J4, Canada; 3 School of Social Work, Faculty of Arts and Sciences, University of Montreal, Montreal, QC H3T 1J4, Canada; 4 Services et Recherches Psychiatriques AD, Montreal, QC H1N 3V2, Canada; kingsada.phraxayavong.cemtl@ssss.gouv.qc.ca; 5 Institut National de Psychiatrie Légale Philippe-Pinel, Montreal, QC H1C 1H1, Canada

**Keywords:** schizophrenia, auditory hallucinations, virtual reality therapy, avatar therapy, sense of presence, emotions

## Abstract

Avatar therapy (AT) is a novel virtual reality-based psychotherapy that has been developed to treat auditory verbal hallucinations (AVH) in treatment-resistant schizophrenia. Various psychotherapeutic components, such as emotions and sense of presence, could contribute to clinical outcomes. However, the interplay between sense of presence, emotions, and clinical response has seldom been investigated. This study aimed to explore the relations between sense of presence, emotions, and clinical outcomes in AT. To conduct this investigation, data from previous and ongoing AT trials were used. Sense of presence and emotions were assessed using standardized questionnaires. AVH were evaluated using the Psychotic Symptom Rating Scales. While sense of presence was positively associated with positive emotions such as control and serenity, no significant associations were found for negative emotions. Moreover, a higher level of sense of presence was associated with a bigger decrease in AVH. Overall, positive emotions seem to be associated with sense of presence in AT. Sense of presence also seems to be involved in the therapeutic outcome, thereby suggesting that this could be an important component related to clinical response. More studies are needed to confirm these trends, which could be generalized to other virtual reality-based psychotherapies.

## 1. Introduction

Schizophrenia is a severe mental illness mainly characterized by delusions, hallucinations, and cognitive dysfunction [1]. This invalidating disorder affects millions of people worldwide and nearly 1% of the general Canadian population, giving rise to considerable societal costs [2,3,4]. Auditory verbal hallucinations (AVH) (i.e., voices heard in the absence of a speaker) are amongst the most debilitating symptoms associated with schizophrenia and are experienced by most individuals with this disorder [5,6]. In fact, AVH have been associated with lower levels of employment and social isolation [7,8].

First-line treatment for people with schizophrenia is based on the administration of antipsychotics [9]. Antipsychotic agents aim to reduce psychotic symptoms [9]. However, up to a third of people with schizophrenia do not respond adequately to these treatments [10]. Clozapine is usually the next recommended pharmacological approach, as it is considered the most effective medication for people with treatment-resistant schizophrenia [11,12,13]. Nonetheless, over 50% of these individuals will not show clinical improvement following this treatment [14]. In these cases, psychological interventions can be combined with pharmacological treatments to optimize outcomes [15]. Moreover, psychological treatments are a valuable option to consider in the treatment of schizophrenia given the significant side effects associated with most antipsychotics [16]. Cognitive-behavioral therapy (CBT) is the most commonly utilized psychotherapy for the treatment of psychotic symptoms [17]. CBT has proven to reduce AVH, but its efficacy remains modest [18].

To address these limitations, novel therapeutic approaches, such as virtual reality (VR)-based therapies, have been put forth. VR has previously been used in a wide array of therapeutic contexts as it can aid in exposing individuals to different stimuli related to their psychopathology and contribute to clinical improvement [19]. Several studies have explored its efficacy for different mental illnesses including anxiety disorders and psychotic disorders [19]. Notably, VR-assisted therapies including CBT components have been shown to improve symptoms such as paranoid ideations in people with schizophrenia disorders [20,21]. Moreover, Avatar therapy (AT) is an innovative VR-based psychotherapeutic intervention which specifically targets AVH [22]. During this intervention, participants communicate with representations of their AVH through a VR interface. Participants are invited to create a computerized visual representation of their most distressful voice and personalize the associated voice [23]. The therapist animates this representation (i.e., the Avatar) and uses it to communicate with the participant. AT allows participants to interact with their voice in a safe environment and eventually develop a sense of empowerment and control. This approach enables the creation of an experience similar to what individuals are faced with when hearing these tormenting voices. Descriptions of the contents of the dialogue with the voices are used by the therapist to recreate realistic interactions with the participant. This helps to explore the participant’s real-life reactions to the voices so that these can be adjusted with the therapist throughout the sessions. Previous trials have demonstrated the effectiveness of AT in reducing the frequency and distress associated with AVH in people with schizophrenia [22,24].

Many aspects of VR, such as sense of presence, have been studied to better understand the role of these components in immersive experiences [25]. Sense of presence is a multidimensional concept generally defined as the sense of being in a context displayed by a virtual environment [26]. Several studies have shown that most virtual environments can induce a sense of presence, which could be linked to emotional reactions and learning [27,28]. In fact, some studies have found a bidirectional relationship between emotions and sense of presence in a therapeutic context, showing that a higher sense of presence is associated with higher emotional intensity and vice versa [29].

Moreover, emotions play a crucial role in psychological therapy efficacy [30,31]. Hence, dialogue during AT can be very emotionally charged. Emotions such as anger, joy, interest, and disgust have been highlighted as being predominant during Avatar–participant interactions [32]. Since emotional response and learning are key components of AT, it is possible that the feeling of presence may impact the clinical outcomes and emotional reactions of participants. Furthermore, some studies have found positive correlations between therapy efficacy for anxiety symptoms and sense of presence [33,34]. One study also found that the interaction between reduction in anxiety and sense of presence was a significant predictor of symptom improvements related to AVH in AT, but that sense of presence alone was not [35]. However, no other studies have examined the mutual interactions between presence, positive and negative emotions, and AVH outcomes in AT.

This exploratory study is one of the first to investigate the relationship between sense of presence, emotional response, and clinical outcomes related to AVH in AT. Based on the previous literature mentioned above, the following was hypothesized: 1. higher levels of sense of presence would correlate positively with higher emotional response and intensity; 2. higher levels of presence would correlate positively with a better clinical outcome; 3. participants with a high clinical improvement would report higher levels of sense of presence than participants with a lesser clinical improvement.

## 2. Materials and Methods

### 2.1. Participants

To conduct this investigation, data from previous and ongoing AT randomized clinical trials were used (ClinicalTrials.gov, accessed on 25 April 2024, identifier numbers: NCT03585127, NCT04054778, and NCT03148639). Participants all provided informed written consent. These trials were approved by the “Centre intégré universitaire de santé et de services sociaux de l’Est-de-l’Île-de-Montréal” ethics committee.

To be included in this study, participants had to be 18 years or older and have a diagnosis of treatment-resistant schizophrenia or schizoaffective disorder, which was defined as the presence of persistent AVH after adequate trials of two or more antipsychotics (therapeutic doses for a minimum of 6 consecutive weeks) [36,37]. Participants had to be stable prior to enrolling in the research projects and able to provide informed consent. Therefore, they were excluded at screening if their medication was changed within the past 6 weeks, if they recently started a new psychotherapy, or if their mental or physical state was fluctuating rapidly. Exclusion criteria included neurocognitive disorders impairing the ability to understand therapy, diagnosis of substance use disorder, and homeless status (for follow-up reasons). Moreover, any significant medication changes during the course of AT led to an exclusion from the project. Clinical diagnoses were confirmed using the Structured Clinical Interview for DSM-5 (SCID-5) [38].

Data from a total of 123 participants with psychotic disorders were included in this study, and 68.3% of the participants were men. While 78.9% of the sample was diagnosed with schizophrenia, 21.1% of the participants suffered from schizoaffective disorder. The detailed sample characteristics are presented in Table 1.

### 2.2. Procedures

Participants had all been enrolled for AT between 2015 and 2023. AT consists of 9 weekly psychotherapeutic sessions including 8 immersive sessions during which participants interact with a virtual representation of their AVH (the Avatar). Participants viewed the Avatar, with a neutral background, through a VR head-mounted display. Each session lasted one hour, with approximately 15 min being allotted to the VR immersion. Before the immersion, the themes the participant wished to address during the immersion were discussed with the therapist. After the immersion, the therapist discussed the participant’s feelings about the immersion and goals for the week to come. The Avatar was created during the first session, based on the participant’s perception of the voice. During the next sessions, participants were encouraged to dialogue with the Avatar and to try different adaptation strategies. The Avatar, which was enacted by the therapist, initially acted in a hostile way toward the participant and repeated what the participant reported hearing from the voices. Indeed, the Avatar used techniques such as threats, accusations, and belittlement to create an experience similar to what the participant experiences with their voice [39]. As the sessions progressed and the participant developed better adaptation strategies with the help of the therapist, the Avatar started to communicate in a more constructive way to encourage the development of the participant’s sense of control and self-esteem. The Avatar used constructive techniques such as reinforcement, active listening, and reconciliation [39]. Moreover, positive behaviors and emotions, such as self-affirmation, have been shown to increase throughout AT sessions as the participants learn how to regulate their negative emotions [39].

Since the first session does not include an immersion, it was not included in the current analysis. The present dataset included participants who had completed between 2 and 8 immersive AT sessions. However, certain participants had participated in more than one trial and therefore completed more than 8 sessions [23]. Additional details regarding AT can be found in one of the preceding trials [22].

### 2.3. Measures

Auditory hallucinations assessments were completed at baseline (around one week before the beginning of AT), post-therapy (around one week after the end of therapy), and follow-up (3 months after the end of AT). These were led by research nurses or research assistants, all of whom underwent questionnaire-specific training before the beginning of the clinical trials. Sociodemographic data were collected during the baseline evaluation. Moreover, during each evaluation, auditory hallucinations were assessed using the auditory hallucinations subscale of the Psychotic Symptom Rating Scales (PSYRATS—AH) [40]. The auditory hallucinations subscale comprises 11 Likert-type items. The PSYRATS was demonstrated to be a reliable instrument for measuring different aspects associated with auditory hallucinations [40]. In the present study, the PSYRATS—AH total score, as well as the following subscales were used for statistical analyses: frequency, distress, attribution, and loudness [41].

The Igroup Presence Questionnaire (IPQ) was used to evaluate sense of presence. This validated questionnaire is composed of 14 items divided into 4 subscales: general presence, spatial presence, involvement, and realism. The items are rated on a 7-point Likert-type scale ranging from 0 (“not at all/fully disagree”) to 6 (“very much/fully agree”). This questionnaire has been previously validated and found to be reliable in different samples, though it has not been validated in a population with psychotic disorders [42]. Participants completed the questionnaire with a research assistant at the end of the first and last immersive sessions, with the exception of 14 participants who completed the IPQ during every immersive session. The IPQ only assessed sense of presence during the immersive session that occurred right before the administration of the questionnaire, and not the previous ones. For the analyses presented in the current paper, a modified version of the IPQ excluding items 3, 11, and 12 was used. Indeed, this change was made due to concerns regarding the participants’ comprehension of certain items. By removing these items, the internal consistency greatly increased; Cronbach’s alpha went from 0.58 to 0.72. This resulted in a total score varying from 6 to 66. Of note, the IPQ was only used during certain trials, therefore less data were acquired with this questionnaire.

Sense of presence was also measured using a graphic rating scale (GRS) ranging from 0 (“not at all”) to 10 (“very strongly”). Participants rated how present they felt with the Avatar during the VR immersion. This question was asked by the therapist at the end of each immersive AT session. This scale was used to mitigate the comprehension concerns raised with the IPQ and include a measure of presence that considers the Avatar.

The emotional response during each therapeutic session was evaluated using a GRS with a rating system ranging from 0 (“not at all”) to 10 (“very strongly”). This scale was utilized to evaluate the following emotions: anxiety, control, anger, fear, serenity, and sadness. Subjects were asked by the therapist to rate how much they felt these emotions during the immersive experience. This questionnaire was completed at the end of each immersive AT session. Since the question regarding sadness was added after the initial AT trials, it was only evaluated in certain subsequent trials, resulting in fewer responses being collected for this emotion.

### 2.4. Statistical Analyses

First, the relation between emotional response and sense of presence was explored with a bivariate Spearman correlation because the data did not present a normal distribution [43]. The GRS scores (sense of presence and emotions) were averaged over all completed immersive AT sessions. IPQ total scores were also averaged over the sessions during which they were completed, thereby providing a representation of the overall sense of presence and emotions experienced throughout AT. The mean scores were used to perform the different correlations. The same procedure was used with the maximum emotion GRS scores for positive emotions (control, serenity), negative emotions (anxiety, anger, fear, sadness), and total emotions. In other words, the highest mean scores in each emotion category (positive, negative, total), regardless of the session, were used for the correlations with sense of presence. These scores were used as a measure for maximum emotional intensity to evaluate the relation between emotional intensity and sense of presence. A bivariate Spearman correlation was also used to evaluate the association between clinical symptomatology and sense of presence. To perform this test, the variation percentage between baseline and 3-month follow-up PSYRATS—AH total scores as well as IPQ average total scores and average scores for the sense of presence GRS were used. PSYRATS—AH score variations were used as an indicator of severity variation in AVH and were calculated using the following formula: ((baseline PSYRATS—AH score—3-month follow-up PSYRATS—AH score)/baseline PSYRATS—AH score × 100). Therefore, a negative result of this variable indicated a reduction in the severity of AVH. If the 3-month follow-up visit had not occurred, post-therapy scores were used instead. The use of 3-month follow-up PSYRATS—AH scores was preferred as clinical symptomatology reaches a higher variation during this period [23]. In fact, more participants tend to experience a reduction of 20% or greater in their psychotic symptoms at the 3-month follow-up, whereas fewer participants show this reduction at the post-therapy evaluation [22,23]. Hence, using the 3-month follow-up evaluation could provide a better understanding regarding the potential association between clinical response and sense of presence. Nevertheless, to verify if the score variations used impacted the results, both (baseline and post-therapy vs. baseline and 3-month follow-up) were compared with a Wilcoxon matched-pairs test [44]. Moreover, the correlations with sense of presence were replicated with each score variation to compare the outcomes (see Table S3). Out of 64 participants who completed baseline and post-therapy PSYRATS—AH evaluations, 39 participants completed the 3-month follow-up PSYRATS—AH evaluation (see Table 1). To carry out clinical outcome-related analyses, data from participants who completed all AT sessions were used.

Next, the relationship between the main therapeutic outcome and sense of presence was explored. Using the previously calculated variation percentages of PSYRATS—AH total scores, a dichotomic analysis was conducted using a reduction threshold of 20% or more in PSYRATS—AH symptomatology as an indicator of a clinical response. This is a commonly used threshold for the evaluation of treatment response in people with schizophrenia [37,45,46]. Thus, participants were categorized in the responders’ group if they had a PSYRATS—AH score reduction of 20% or more. Otherwise, they were categorized in the non-responders’ group. A total of 18 participants were categorized in the responders’ group and 46 in the non-responders’ group (see Table 1). A Mann–Whitney U test was then used to compare average sense of presence scores (GRS mean scores and IPQ mean scores) between responders and non-responders [47]. The effect size for this test was estimated with Cohen’s d [48].

Afterwards, associations between sense of presence (IPQ mean scores and GRS mean scores) and PSYRATS—AH subscale score variation percentages were evaluated using bivariate Spearman correlations (see Table S1). PSYRATS—AH subscale score variation percentages were calculated using the baseline PSYRATS—AH subscale scores and the 3-month follow-up PSYRATS—AH subscale scores, or the post-therapy PSYRATS—AH subscale scores when 3-month follow-up scores were not available. Also, the relationship between mean emotional response scores and average PSYRATS—AH subscale score variation percentages was assessed using bivariate Spearman correlations (see Table S2).

Finally, to evaluate the potential influence of age and sex on the different variables, results were compared between male and female participants, and age was correlated with all the variables (sense of presence, emotions, emotional intensity, and PSYRATS—AH score variations) (see Tables S4 and S5).

As some data from different questionnaires had not yet been collected for participants from ongoing trials, or had not been collected in previous trials, available measures sometimes varied between statistical tests. Statistical analyses were carried out using SPSS 28 [49]. Correlation coefficients were categorized as weak (±0.1–0.3), moderate (±0.4–0.6), strong (±0.7–0.9), and perfect (±1) [50]. Effect sizes (Cohen’s d) were categorized as small (0.2–0.5), moderate (0.5–0.8), and large (>0.8) [51]. The *p*-value significance threshold was set to 0.05.

## 3. Results

### 3.1. Sense of Presence and Emotions 

**Figure 1:**
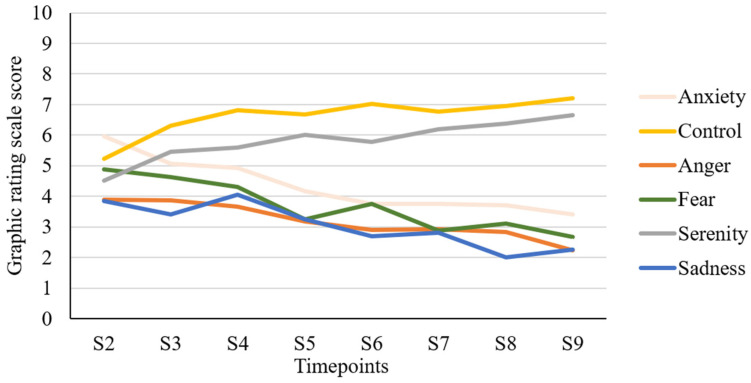
Estimated means of emotion scores over Avatar therapy sessions (N = 112). N: Sample size; S: Session.

The evolution of emotional response scores throughout the sessions is illustrated in Figure 1. Emotions related to control and serenity tended to increase throughout the therapy sessions, while feelings of anxiety, fear, anger, and sadness tended to decrease.

Regarding the association between the two presence scales, a moderate positive correlation was observed between the GRS mean scores and IPQ mean scores (r_s_ = 0.460, *p* < 0.01). The impact of presence on experienced emotions during AT is detailed in Table 2. A weak to moderate positive correlation between sense of presence and control (r_s_ = 0.337, *p* < 0.01), and a weak positive correlation between sense of presence and serenity (r_s_ = 0.242, *p* < 0.05) were detected with the GRS scores. Also, a weak positive correlation between sense of presence and control (r_s_ = 0.225, *p* < 0.05) was detected with the IPQ scores. However, no significant correlation was found between presence and serenity with the IPQ scores (r_s_ = 0.113, *p* = 0.305).

The impact of presence on emotional intensity during AT is presented in Table 2. With the GRS scores, a weak to moderate positive correlation between sense of presence and positive emotional intensity (r_s_ = 0.337, *p* < 0.01), and a weak positive correlation between sense of presence and total emotional intensity (r_s_ = 0.242, *p* < 0.05) were observed. Regarding the IPQ scores, a weak positive correlation was found between sense of presence and total emotional intensity (r_s_ = 0.284, *p* < 0.01).

### 3.2. Sense of Presence and Clinical Outcomes

The results regarding the impact of presence on variation percentages in PSYRATS—AH scores were mixed (as shown in Table 2). In fact, with the GRS scores, sense of presence was weakly to moderately associated with a reduction in auditory hallucinations (r_s_ = −0.384, *p* < 0.01). In other words, higher levels of presence were correlated with a reduction in AVH. However, with the IPQ scores, no significant correlation was found between these variables (r_s_ = −0.257, *p* = 0.15).

The PSYRATS-AH score variations calculated with 3-month follow-up scores did not differ from those calculated with the post-therapy scores (Z = 269.5, *p* = 0.632). When score variations were correlated separately with presence, higher sense of presence scores were associated with a reduction in auditory hallucinations at the post-therapy evaluation (GRS: r_s_ = −0.485, *p* = 0.019; IPQ: r_s_ = −0.464, *p* = 0.039). No associations were observed with the 3-month follow-up score variations (see Table S3).

Then, mixed results were found concerning sense of presence score differences between responders and non-responders (as shown in Table 3). The GRS mean scores showed a significant difference between sense of presence scores in the two groups (*p* = 0.047), with a moderate effect size (d = 0.543). In fact, with the GRS, participants in the responders’ group had higher sense of presence scores. However, with the IPQ scores, no significant difference between sense of presence scores was detected (*p* = 0.963).

Higher GRS presence scores were associated with a higher decrease in distress PSYRATS—AH subscale scores, and a higher decrease in loudness PSYRATS—AH subscale scores. Greater IPQ scores were also correlated with a greater decrease in loudness PSYRATS—AH subscale scores (see Table S1). Higher levels of certain negative emotions (anger, anxiety, maximum intensity of negative emotions) were associated with a higher reduction in frequency PSYRATS—AH subscale scores (see Table S2). No significant correlations were noted between the other PSYRATS—AH subscales (distress, attribution, loudness) and emotions (See Table S2).

Overall, the results did not differ between male and female participants. However, a weak negative correlation was observed between GRS mean scores and anger among male participants (r_s_ = −0.283, *p* = 0.019) (see Table S4). Age seemed to have a minimal effect on most associations. Indeed, a weak positive association (r_s_ = 0.191, *p* = 0.043) was found between age and emotional intensity for total emotions, but no other associations were observed (see Table S5).

## 4. Discussion

This study sought to explore the relationships between sense of presence, emotions, and clinical outcomes in AT. Positive associations between sense of presence and control were found. A positive association was also found between sense of presence and serenity. Moreover, positive associations between sense of presence and emotional intensity were also observed. Relative to the results for the relationship between therapeutic outcome and sense of presence, the two sense of presence questionnaires (IPQ and GRS) yielded different outcomes. Indeed, higher GRS presence scores were associated with a reduction in AVH and a better clinical outcome. However, those associations were not observed with the IPQ scores, except when they were correlated exclusively with the variation in AVH observed at the post-therapy evaluation. 

Regarding the evolution of emotional responses during AT, the results suggest that positive emotions (control, serenity) became more prominent towards the end of AT. Inversely, negative emotions (anger, fear, anxiety, sadness) seemed to decrease as the sessions progressed. Similarly, previous studies have demonstrated that levels of anxiety and fear tend to diminish significantly between the first and last session of AT [23,35]. Traditional interventions for psychotic disorders, such as acceptance and commitment therapy, have also shown that feelings of anxiety tend to diminish during the therapeutic process [52]. Moreover, the noted increase in positive emotions aligns with one of the therapeutic goals of AT, which is to help participants develop a sense of control over the voices they hear and reduce the distressing feelings associated with them. Overall, these results suggest that AT could enhance positive emotions while reducing negative emotions.

In addition, sense of presence, evaluated with the GRS, was positively correlated with positive emotions. Similar results were observed with the IPQ, as the IPQ scores were positively correlated with control. This is congruent with the moderate positive association found between the two scales, which indicates that there is some overlap between the measures of sense of presence reported by the GRS and the IPQ. The results regarding sense of presence and emotional intensity are coherent with these outcomes. In fact, the sense of presence GRS and IPQ scores were both positively associated with emotional intensity for total emotions, which indicates that emotional response intensity is directly associated with levels of sense of presence in AT. Consistent with these results, previous literature has shown that emotions may be a fundamental factor influencing sense of presence [29,53]. Additionally, GRS presence scores were positively correlated with emotional intensity for positive emotions, which, combined with the previous results, suggests that sense of presence could be more strongly associated with positive emotions, compared to negative emotions, in AT. Other VR-based therapy studies have mostly explored the relationship between negative emotions and sense of presence, showing that there seems to be a positive association between these two variables [54]. However, these VR interventions are predominantly targeted towards phobias and mostly involve an approach using exposure therapy [54]. This may contribute to explaining the disparities with the present findings [54]. Moreover, as AT progresses, the Avatar tends to act in a more positive and friendly way toward the participant compared to the first sessions during which the Avatar will act in a negative and confrontational manner with the participant [39]. This is congruent with the previous results showing that positive emotions become more important as the therapy sessions progress. This implies that positive emotions could be a central aspect of the Avatar–participant dynamic, which could explain why associations with sense of presence were mainly observed with positive emotions. Freeman et al. (2005) have previously proposed that the relationship between presence and emotions could be dependent on the stimulus and its significance for the viewer [55]. Therefore, associations between emotions and sense of presence may vary based on the dynamics involved in the VR intervention. Nevertheless, more studies are needed to further understand the associations between emotions and presence in AT and other VR-based therapies for psychotic disorders.

As for the associations between the sense of presence GRS scores and the PSYRATS—AH score variations, these results suggest that the reduction in severity of AVH is associated with a higher sense of presence. Also, the variations between PSYRATS-AH post-therapy scores and baseline scores were correlated with both the sense of presence GRS scores and the IPQ scores. This suggests that presence is more strongly associated with the reduction in AVH severity at the post-therapy evaluation compared to the 3-month follow-up. Other VR-based therapy studies show conflicting results regarding correlations between sense of presence and clinical outcomes. Indeed, some studies have shown that presence is significantly associated with therapeutic outcomes in interventions targeting anxiety and phobias [34,56]. However, other anxiety and phobia studies have found no association between these variables [33]. Further research is needed to better understand the association between presence and clinical outcomes. Furthermore, the sense of presence GRS scores were significantly higher for AT responders compared to non-responders, which is congruent with the association found between the reduction in AVH symptomatology and higher GRS presence scores. However, these results were not observed with the IPQ scores.

Collectively, these results seem to indicate that sense of presence may be a contributing factor to clinical outcomes in AT. Despite some associations with the sense of presence GRS scores and the IPQ scores being similar, it is possible that the differences regarding therapeutic outcomes are attributable to variations in measures between the two scales. In fact, even though a significant positive correlation between the sense of presence GRS scores and the IPQ scores was found, this association remains moderate. The GRS involves a relational measure of presence, as it takes into consideration the Avatar. Conversely, the IPQ mainly evaluates physical presence in the VR environment [42]. This measure may be complexified with the use of a neutral VR background in AT, as the characteristics of VR environments may impact presence. Since AT is a psychological intervention centered on the relationship the participant has with the Avatar, it is possible that the consideration of the relational aspect of presence, with the GRS, may have impacted sense of presence scores. Moreover, the IPQ has not yet been validated in people suffering from psychotic disorders and may therefore not be adapted for this population. Indeed, this questionnaire may be too lengthy and complex for this population, which could explain why the internal consistency for the IPQ was lower before the removal of some of the questions. Considering the difference in results between the two scales, developing sense of presence scales validated in populations with psychotic disorders could be beneficial.

The negative association between anger and the GRS scores for male participants suggests that negative emotions could be inversely correlated with presence in AT, contradicting findings from most VR-based therapy studies. While this association was not observed in female participants, this difference is likely due to the limited number of females in the sample. Future studies with larger samples should further investigate the possible sex differences in associations between presence and emotions in AT. Additionally, age may have influenced the associations between sense of presence and emotional intensity for total emotions. However, because the association observed was weak, this impact was probably negligible.

Though this study provides a new insight into the role of sense of presence and emotions in AT, some limitations should be considered while interpreting the results of this investigation. Firstly, the sense of presence GRS has not been previously validated. To compensate for this limit, the IPQ was also used, which allowed for the comparison of the two scales. Secondly, the number of participants was limited. Thus, a limited statistical power could have led to the underestimation of certain results, especially regarding the correlations between presence and sadness where the sample size was lower compared to the correlations with other emotions. Nonetheless, multiple associations were statistically significant. Thirdly, the number of completed AT sessions varied between participants, which may have affected the mean emotional response scores, as therapeutic content varied between AT sessions, thereby affecting emotional response. Future studies, with larger sample sizes, should explore how associations between presence and emotions are affected by the number of sessions completed. Finally, as this is a correlational study, causal relationships cannot be established between the analyzed variables. It is also important to note that, because this is an exploratory study, these results cannot be generalized, which limits their interpretation.

## 5. Conclusions

In conclusion, this study’s main objective was to evaluate the relationship between sense of presence, emotional response, and clinical outcomes in AT. It was found that sense of presence is mainly associated with positive emotions and overall emotional intensity. Additionally, it was observed that greater levels of presence may contribute to the improvement of AVH. Since VR interventions for psychotic disorders are newly emerging, it is important to understand the role sense of presence plays in the effectiveness of such therapies. It is also important to explore the role of emotional responses in VR-based therapies, as emotions are a central part of psychological interventions. Furthermore, adding objective measures of sense of presence and a presence questionnaire centered on the social aspect of presence could help to obtain measures more specific to AT’s therapeutic objectives. However, other studies are needed to verify the reproducibility of these results in AT, but also in other VR-based psychotherapeutic interventions for psychotic disorders.

## Figures and Tables

**Table 1:** Sample characteristics (N = 123).

Characteristics	Mean or N	SD or %
Age, years	41.9	12.9
Male sex	84	68.3%
Ethnicity		
Caucasian	96	78.0%
African American	9	8.1%
Latin American	3	2.4%
Other	14	11.4%
Highest educational attainment		
Less than high school	38	30.9%
High school diploma	23	18.7%
Postsecondary education	62	50.4%
Diagnosis		
Schizophrenia	97	78.9%
Schizoaffective disorder	26	21.1%
Clinical response (≥20% reduction in hallucinations, N = 64)		
Responders	18	28.1%
Non-responders	46	71.9%
Clinical response evaluation based on		
Post-therapy evaluation	25	39.1%
3-month follow-up evaluation	39	60.9%

N: sample size; SD: Standard deviation; %: percentage.

**Table 2:** Correlations between sense of presence, evaluated with the GRS and the IPQ, and emotions, emotional intensity, and variation percentage of PSYRATS—AH score.

	GRS Mean Scores			IPQ Mean Scores		
	N	r_s_	*p*-Value	N	r_s_	*p*-Value
**Mean Emotional Response**						
Sadness	68	−0.033	0.790	67	0.093	0.452
Anger	112	−0.151	0.112	84	0.085	0.442
Anxiety	112	−0.092	0.333	84	0.039	0.725
Fear	112	−0.173	0.067	84	0.113	0.306
Serenity	112	0.242 *	0.010	84	0.113	0.305
Control	112	0.337 **	<0.001	84	0.225 *	0.040
**Maximal emotional intensity**						
Positive	112	0.355 **	<0.001	84	0.209	0.056
Negative	112	−0.153	0.107	84	0.097	0.381
Total	112	0.398 **	<0.001	84	0.284 **	0.009
**Variation in the severity of auditory hallucinations (3-month follow-up vs. baseline)**						
PSYRATS—AH (%)	60	−0.384 **	0.002	33	−0.257	0.149

GRS: graphic rating scale; IPQ: Igroup Presence Questionnaire; N: sample size; r_s_: Spearman correlation coefficient; %: percentage; PSYRATS—AH: Psychotic Symptom Rating Scales—Auditory Hallucinations. Note: Variation percentages were calculated using baseline PSYRAT—AH scores and 3-month follow-up PSYRAT—AH scores, or, when 3-month follow-up PSYRAT—AH scores were not available, post-therapy PSYRATS—AH scores were used. A negative variation indicated a reduction in psychotic symptoms following therapy.

**Table 3:** Comparisons of sense of presence scores between responders and non-responders to AT.

	N	Responders Mean Score (SD)	Non-Responders Mean Score (SD)	*p*-Value	Cohen’s d
GRS	60	8.9 (3.5)	7.4 (1.4)	0.047	0.543
IPQ	33	42.1 (7.0)	41.8 (9.3)	0.963	0.034

AT: Avatar therapy; GRS: graphic rating scale; IPQ: Igroup Presence Questionnaire; SD: standard deviation; N: sample size.

## Data Availability

The datasets presented in this article are not readily available because the data are part of an ongoing study. Requests to access the datasets should be directed to Dr. Alexandre Dumais.

## Supplementary Materials

The following supporting information can be downloaded at: https://www.mdpi.com/article/10.3390/jpm14060614/s1, Table S1: Correlations between the variation percentages of PSYRATS—AH subscale scores and sense of presence evaluated with the GRS and the IPQ; Table S2: Correlations between the variation percentages of PSYRATS—AH subscale scores and emotions; Table S3. Correlations between sense of presence, evaluated with the GRS and the IPQ, and variation percentages of PSYRATS—AH scores (3-month follow-up vs baseline and post-therapy vs baseline); Table S4. Correlations between sense of presence, evaluated with the GRS and the IPQ, and emotions, emotional intensity, and variation percentage of PSYRATS—AH scores for male and female participants; Table S5. Correlations between age and sense of presence, evaluated with the GRS and the IPQ, emotions, emotional intensity, and variation percentage of PSYRATS—AH scores.
