# Supplementary material for: The Relationship between Sense of Presence, Emotional Response, and Clinical Outcomes in Virtual Reality-Based Therapy for Treatment-Resistant Schizophrenia: An Exploratory Correlational Study
Source: J Pers Med. 2024 Jun 8;14(6):614. doi: 10.3390/jpm14060614 (PMC11204393; doi:10.3390/jpm14060614)
Supplement: Supplementary file 1 [file jpm-14-00614-s001.zip › jpm-3011097-supplementary.pdf]

Supplementary tables

Note: Variation percentages were calculated using baseline PSYRATS—AH scores and 3-month follow-up PSYRATS—AH scores, or when 3-month follow-up PSYRATS—AH scores were not available, post-therapy PSYRATS—AH scores were used. A negative result of PSYRATS—AH variation percentage indicated a reduction in the severity of auditory hallucinations.

Table S1. Correlations between the variation percentages of PSYRATS – AH subscale scores and sense of presence evaluated with the GRS and the IPQ.

	**GRS mean scores (N=60)**	**IPQ mean scores (N=33)**
**PSYRATS – AH subscales**	r_s	r_s
Attribution	-0.138	-0.095
Distress	-0.310*	0.047
Frequency	0.225	-0.331
Loudness	-0.285*	-0.402*

GRS: graphic rating scale; IPQ: Igroup Presence Questionnaire; PSYRATS—AH: Psychotic Symptom Rating Scales – Auditory Hallucinations; N: sample size; r_s: Spearman correlation coefficient.
**p < 0.01
*p <0.05

Table S2. Correlations between the variation percentages of PSYRATS—AH subscale scores and emotions.

		**PSYRATS—AH Attribution**	**PSYRATS—AH Distress**	**PSYRATS—AH Frequency**	**PSYRATS—AH Loudness**
**Mean emotional response**	**N**	r_s	r_s	r_s	r_s
Sadness	18	-0.272	0.051	-0.241	0.289
Anger	60	-0.088	-0.044	-0.290*	0.153
Anxiety	60	-0.085	0.107	-0.343**	-0.017
Control	60	0.041	-0.216	-0.003	-0.109
Fear	60	-0.065	0.198	-0.233	-0.080
Serenity	60	0.191	-0.115	0.097	-0.092
Maximum of negative emotions	60	-0.092	0.116	-0.314*	0.028

PSYRATS—AH: Psychotic Symptom Rating Scales – Auditory Hallucinations; N: sample size; r_s: Spearman correlation coefficient.
**p < 0.01
*p <0.05

Table S3. Correlations between sense of presence, evaluated with the GRS and the IPQ, and variation percentages of PSYRATS—AH scores (3-month follow-up vs baseline and post-therapy vs baseline).

	**GRS mean scores**			**IPQ mean scores**		
	**N**	r_s	**p-value**	**N**	r_s	**p-value**
**Variation in the severity of auditory hallucinations**						
PSYRATS—AH (%) **(3-month follow-up vs. baseline)**	37	-0.307	0.065	13	-0.041	0.894
PSYRATS—AH (%) **(post-therapy vs. baseline)**	23	-0.485*	0.019	20	-0.464*	0.039

GRS: graphic rating scale; IPQ: Igroup Presence Questionnaire; PSYRATS—AH: Psychotic Symptom Rating Scales – Auditory Hallucinations; N: sample size; r_s: Spearman correlation coefficient.

Table S4. Correlations between sense of presence, evaluated with the GRS and the IPQ, and emotions, emotional intensity, and variation percentage of PSYRATS—AH scores for male and female participants.

	**Male participants**						**Female participants**					
	**GRS mean scores**			**IPQ mean scores**			**GRS mean scores**			**IPQ mean scores**		
	**N**	r_s	**p-value**	**N**	r_s	**p-value**	**N**	r_s	**p-value**	**N**	r_s	**p-value**
**Mean emotional response**												
Sadness	42	-0.113	0.475	41	0.065	0.686	26	0.170	0.407	26	0.139	0.499
Anger	76	-0.283*	0.013	55	0.019	0.892	36	0.126	0.464	29	0.192	0.318
Anxiety	76	-0.102	0.381	55	-0.084	0.541	36	-0.070	0.684	29	0.227	0.235
Fear	76	-0.151	0.194	55	0.057	0.682	36	-0.223	0.190	29	0.217	0.258
Serenity	76	0.341**	0.003	55	0.101	0.462	36	0.031	0.856	29	0.164	0.394
Control	76	0.272*	0.017	55	0.182	0.185	36	0.448**	0.006	29	0.345	0.067
**Maximal emotional intensity**												
Positive	76	0.363**	0.001	55	0.170	0.216	36	0.305	0.070	29	0.342	0.070
Negative	76	-0.207	0.073	55	-0.008	0.951	36	-0.007	0.968	29	0.249	0.193
Total	76	0.366**	0.001	55	0.280*	0.038	36	0.449**	0.006	29	0.300	0.114
**Variation in the severity of auditory hallucinations (3-month follow-up vs. baseline)**												
PSYRATS—AH (%)	45	-0.418**	0.004	25	-0.115	0.585	15	-0.334	0.224	8	-0.476	0.233

GRS: graphic rating scale; IPQ: Igroup Presence Questionnaire; PSYRATS—AH: Psychotic Symptom Rating Scales – Auditory Hallucinations; N: sample size; r_s: Spearman correlation coefficient.

Table S5. Correlations between age and sense of presence, evaluated with the GRS and the IPQ, emotions, emotional intensity, and variation percentage of PSYRATS—AH scores.

	Age		
	**N**	**r_s**	**p-value**
**Mean emotional response**			
Sadness	68	-0.014	0.911
Anger	112	0.077	0.417
Anxiety	112	-0.032	0.735
Fear	112	0.055	0.561
Serenity	112	0.037	0.697
Control	112	-0.032	0.646
**Maximal emotional intensity**			
Positive	112	0.043	0.652
Negative	112	0.041	0.669
Total	112	0.191*	0.043
**Sense of presence**			
GRS mean scores	112	0.136	0.153
IPQ mean scores	84	0.207	0.059
**Variation in the severity of auditory hallucinations (3-month follow-up vs. baseline)**			
PSYRATS—AH (%)	64	-0.173	0.171

GRS: graphic rating scale; IPQ: Igroup Presence Questionnaire; PSYRATS—AH: Psychotic Symptom Rating Scales – Auditory Hallucinations; N: sample size; r_s: Spearman correlation coefficient.
